# Self-Perceived Pain in Chinese Patients With Cancer

**DOI:** 10.3389/fpsyg.2019.01994

**Published:** 2019-08-28

**Authors:** Yongfu Zhang, Xiaomin Tan, Wengao Li, Hongmei Wang, Hengwen Sun, Ting Liu, Jingying Zhang, Bin Zhang, Yuan Yang

**Affiliations:** ^1^Department of Anesthesiology, Guangzhou Women and Children’s Medical Center, Guangzhou, China; ^2^Department of Educational Administration, Nanfang Hospital, Southern Medical University, Guangzhou, China; ^3^Department of Psychiatry, Nanfang Hospital, Southern Medical University, Guangzhou, China; ^4^Department of Radiotherapy, Nanfang Hospital, Southern Medical University, Guangzhou, China; ^5^Department of Radiation, Cancer Center of Guangdong Provincial People’s Hospital, Guangdong Academy of Medical Sciences, Guangzhou, China

**Keywords:** cancer, Chinese, clinical, demographic, factor, pain, psychological

## Abstract

**Background:**

Pain is one of the most burdensome and prevalent symptoms cancer patient report and it has severe negative impact on patient’s quality of life. The aim of this study is to estimate the prevalence of pain and to test the association between demographic, clinical, psychological factors, and self-assessed pain in Chinese cancer population.

**Methods:**

A total of 553 cancer patients were recruited in this cross-sectional study. Patient’s basic demographic data was collected by a study-designed information sheet, and patient’s pain, sleep disturbance and psychological distress were assessed by several validated measurements (MPQ-SF, AIS, FoP-Q-SF, PHQ-9, and GAD-7). Descriptive statistics and hierarchical multiple regression analyses were performed.

**Results:**

Of the 553 patients, 411 (74.32%) patients reported that they experience some degree of pain. Fear of progression, anxiety, insomnia, and depressive symptoms were significantly associated with different subscales and the overall pain score in bivariate correlation matrix. Insomnia, depressive symptoms, and fear of cancer progression were significant independent factors of cancer pain on multivariate analyses.

**Conclusion:**

Psychological factors play a great role in the relationship between objective pathophysiology and patient’s subjective experience of pain. It is important to evaluate each individual in detail with respect to psychological distress and pain severity when planning treatment and rehabilitation.

## Introduction

Suffering comes in many ways for cancer patients, and pain is one of the most burdensome and prevalent symptoms they report. Over 30% of cancer patients describe their pain as distressing or even intolerable (Breivik et al., 2009). Cancer treatment such as surgery, radiotherapy, and chemotherapy cause significant acute and chronic pain (Miller et al., 2018). It has been reported that nearly half of cancer patients experience pain, and about 40% of cancer survivors develop chronic pain (Miller et al., 2018). A western meta-analysis in 2016 reported that pain prevalence rates were 55% during cancer treatment, 39.3% post treatment, and 66.4% in metastatic or terminal disease (van den Beuken-van Everdingen et al., 2016). In mainland China, 49.8–80% of cancer patients report some degree of pain, and 11–30% of them reported high level of pain (Wang, 2006; Chen, 2007; Liang, 2016; Tang, 2017).

Pain has severe negative influence on cancer patient’s quality of life (QoL). Patients often report that pain prevents them from focusing or thinking, and that it makes difficulty in performing normal daily activities (Breivik et al., 2009). Studies also found significant relationship between persistent pain experience and emotional connectedness difficulties, and this relationship was mediated by psychological distress (Henne et al., 2015). Thus, researchers highlighted the importance of providing adequate pain treatment which may help to result in clinically improvements in health-related QoL (Puetzler et al., 2014).

Pain is influenced by several physiological, psychological, and behavioral factors (Potter et al., 2014). Studies found that patients with higher levels of psychological distress tended to report greater self-perceived pain as well as decreased self-perceived function (Block et al., 2001; Chaichana et al., 2011), and anxiety, depression, and insomnia symptoms were consistently found to be correlated with the severity of pain (Fishbain et al., 1997; Wilson et al., 2002; Davison and Jhangri, 2005). Additionally, a recent review concluded that depression was a possible predictor in the transition from acute to chronic pain in non-cancer patients (Hruschak and Cochran, 2018). Besides depression, this review also suggested that “fear-avoidance” beliefs in the form of cognitive expectances was another significant predictor in chronicity (Hruschak and Cochran, 2018). However, these factors have not been thoroughly investigated in cancer populations. As one of the most prevalent emotional burdens for cancer patients, fear of cancer progression (FoP) has received increasing attention in recent years. Significant negative associations were found between FoP and medicine adherence, QoL and patient’s psychosocial well-being (Dinkel et al., 2014), and higher FoP was consistently found to be related to more physical symptoms (Koch et al., 2013). However, to the author’s best knowledge, no study has previously investigated the association of FoP and cancer pain.

As suggested by the biopsychosocial models, an increasing number of researchers have delineated psychosocial variables to be important correlates of pain intensity (Vranceanu et al., 2009). However, data on pain prevalence and its influencing factors in Chinese cancer populations are limited, and those studies were all published in Chinese and the majority of them only included small sample size and used single-item question to assess pain. Liang (2016) found that gender, education background, cancer stage, as well as type of painkiller significantly influenced patient’s pain levels, while Chen’s study indicated that lower family income, higher levels of anxiety and depression were associated with severer pain (Chen, 2007). A new study in 2018 suggested that patient’s age, self-efficacy, and coping strategy significantly predicted a cancer patient’s self-perceived pain (Chen, 2018).

In the current study, we aim to (1) estimate the prevalence of pain in Chinese cancer population using validated measurements; (2) test whether demographic, clinical as well as psychological factors are correlated with cancer pain; and (3) assess the relevance between pain and FoP. We hypothesize that: (1) compared to demographic and clinical variables, psychological factors play greater role in patient’s self-perceived pain; (2) anxiety, depression, and insomnia symptoms are positively associated with cancer pain; and (3) patients who report higher FoP are more likely to report greater pain.

## Materials and Methods

### Study Design and Setting

This is a cross-sectional study that involved three main hospitals in southern China. A power analysis was performed before recruitment to determine study sample size. All participants were recruited from the department of oncology (Nanfang Hospital), department of breast surgery (Guangzhou Women and Children’s Medical Center), and department of radiotherapy (Guangdong Provincial People’s Hospital) from January to October 2018. To be included in the study, participants needed to: (1) be above 18 years old; (2) had a clear diagnosis of cancer; (3) be able to read and write Mandarin or Cantonese. Participants were excluded if they had disturbance of consciousness, blind, or deaf.

### Measures

#### Demographic and Clinical Data

Patient’s basic demographic (i.e., age, gender etc.) and clinical (i.e., cancer site, cancer stage etc.) data were collected by a specific question sheet. Information about patient’s treatment received (chemotherapy and radiotherapy), smoking behavior, and physical comorbidity were assessed by “Yes/No” questions. Patient’s personality tendency was measured by a single question derived from the Chinese version Personality Traits Questionnaire (Yan, 2013): what do you think is your dominant personality trait (Positive or Negative)? For patients who were uncertain about their disease stage, information was collected from the individual’s medical notes.

#### The McGill Pain Questionnaire-Short Form (MPQ-SF)

The MPQ-SF (Melzack, 1987) is one of the most widely used tests for the measurement of pain and has been validated into Chinese language in 2013. The Chinese version of MPQ-SF has 17 items in total which consists of three subscales: pain rating index (PRI, item 1–15), visual analog scale (VAS, item 16), and present pain intensity (PPI, item 17). Item 1–11 represent the sensory dimension (PRI-sensory) of pain experience and item 12–15 represent the affective dimension (PRI-affective). These 15 PRI descriptors are rated on a Likert-type scale as 0 = none, 1 = mild, 2 = moderate, and 3 = severe, while the score of VAS (item 16) ranges from 0 (no pain) to 100 (worst possible pain), and the PPI (item 17) score ranges from 0 (no pain) to 5 (excruciating) (Peng and Zhang, 2013).

#### Athens Insomnia Scale (AIS)

The AIS (Soldatos et al., 2000) is commonly utilized in clinical practice as an instrument to assess the intensity of sleep-related problems, and it is also used as a screener in reliably establishing the diagnosis of insomnia. The AIS includes eight items. The first five are related to nocturnal sleep and the last three items are related to daytime dysfunction. All of the items are scored on a Likert-type scale ranging from 0 (no problem/none) to 3 (serious problem), and an individual’s sleep outcome is finally evaluated by the sum score of all items. The Cronbach’s alpha of AIS is 0.89 which shows satisfactory internal consistency. A cut-off of ≥6 on the AIS is used to assist the diagnosis of insomnia (Soldatos et al., 2003).

#### Fear of Progression Questionnaire-Short Form (FoP-Q-SF)

The FoP-Q-SF is a 12-item scale used to assess cancer patient’s fear of progression. It is the short form of the original 43-item Fear of Progression Questionnaire (FoP-Q) (Herschbach et al., 2005). All of the items are rated on a 5-point Likert scale, ranging from 1 (never) to 5 (very often). The resulting cumulative score of the FoP-Q-SF ranges from 12 to 60, and a score of 34 or above indicates a dysfunctional level of FoP (Herschbach et al., 2010). The psychometric properties of the Chinese FoP-Q-SF is satisfactory (Cronbach’s alpha = 0.883) (Wu et al., 2015).

#### Patient Health Questionnaire (PHQ-9)

The PHQ is a 9-item self-assessed measure that evaluates the degree of an individual’s depressive symptoms. The item of PHQ ranges from 0 (not at all) to 3 (almost everyday) (Kroenke et al., 2010) and it has been validated in several specific clinical populations. The Chinese version of the scale showed excellent psychometric properties (internal consistency = 0.89) (Chen et al., 2015). A total score of ≥5 on the PHQ-9 indicated depressive symptoms.

#### General Anxiety Disorder Scale (GAD-7)

The GAD is a 7-item scale used to measure a person’s anxiety level. Response options are not at all, several days, more than half the days, and nearly every day, rated as 0, 1, 2, and 3, respectively (Spitzer et al., 2006). The original internal consistency of the scale was good (Cronbach’s alpha = 0.92), and the Chinese version’s Cronbach’s alpha was 0.91 (Zheng, 2013). A sum score of 5 or more indicated anxiety symptoms.

#### The Holmes-Rahe Life Stress Inventory

The Life Stress Inventory was developed to test the relationship between illness and stress (Holmes and Rahe, 1967). It is also commonly known as the Social Readjustment Rating Scale (SRRS) and has been validated in Chinese (Yang and Zhang, 1999). Forty-three major life stressors (such as death of a spouse/child, divorce, and retirement, etc.) were listed to test an individual’s stress level. The more of the stressors one individual experienced, the higher risk of illness.

### Data Collection

Patients were consecutively recruited from three hospitals. All patients were approached in the waiting room before their appointment with their oncologist and were asked for participation. For those who showed interest in participating, a written informed consent form was provided, then they were asked to complete the information sheet and all scales. Participants were ensured that their personal information would be kept confidential and they could withdraw their participation at any time. The hospital Research Ethics Committee examined and approved the study (Ref No: NFEC-2018-038).

### Data Analysis

Statistical analyses were preformed using SPSS version 22.0 and the cutoff for statistical significance was set at 0.05 (2 sided). Descriptive statistics were used to calculate frequencies, means, and standard deviations. Difference in means were investigated using a *t* test for independent samples. Analysis of variance and *post hoc* test were used to identify group effects. Continuous variables that were not normally distributed were analyzed using Mann-Whitney U tests or Kruskal-Wallis tests (K-W tests), as appropriate. The associations between continuous variables were assessed using Pearson’s correlation analysis if the variables were normally distributed, otherwise, Spearman’s correlation was utilized. Hierarchical regression analyses were subsequently conducted to ascertain the associated factors of cancer pain (three subscales: PRI, VAS, and PPI). Three blocks were orderly included in each regression analyses. Demographic factors were entered in the first block, followed by the clinical/treatment factors, and the final block involved the psychological variables.

## Results

### Patient Characteristics

A total of 887 patients were invited to participate the study and 566 (63.81%) of them agreed. Thirteen patients were then excluded from the study because they were teenagers (age under 18 years). Finally, 553 patients completed the survey. The mean age of patients is 47.23 (SD = 11.26, ranged from 23 to 90). The majority of the participants were female (91.7%), married (87.5%), diagnosed with breast cancer (84.6%), non-current smoker (96.2%) and had received chemotherapy (90.6%), and radiotherapy (86.6%). All participants were currently receiving active treatment (radiation/chemotherapy/drugs). Less than half of the participants experienced clinical insomnia (40.7%), FoP (29.8%), depressive or anxiety symptoms (42.0 and 30.7%, respectively). Of the 553 participants, 142 (25.68%) patients reported no pain (total score = 0), and the remaining 411 (74.32%) patients reported some degree of pain. Patient’s demographic, clinical as well as psychological characteristics are presented in Table 1.

**TABLE 1:** Demographic, clinical, and psychological characteristics of participants (*N* = 553).

		**PRI-Total**		**VAS**		**PPI**	
	***n*** **(%)**	**M ± SD**	**Z^b/c^**	**M ± SD**	**Z^b/c^**	**M ± SD**	**T/F**
**Sociodemographic**							
**Gender**							
Male	46 (8.3)	5.67 ± 7.65		24.78 ± 17.73		1.57 ± 0.69	
Female	507 (91.7)	5.10 ± 5.92	–0.508	25.21 ± 17.36	–0.235	1.64 ± 0.76	–0.618
**Age**							
Age below 35	71 (12.8)	4.83 ± 4.48		25.77 ± 16.96		1.59 ± 0.73	
Age between 35-60	391 (70.7)	4.82 ± 6.09		23.73 ± 16.51		1.62 ± 0.77	
Age above 60	91 (16.5)	6.80 ± 6.82	8.201^∗^	30.88 ± 20.09	11.807^∗∗^	1.71 ± 0.69	0.700
**Marital Status**							
Single	32 (5.8)	5.78 ± 4.80		27.50 ± 19.84		1.75 ± 0.76	
Married	484 (87.5)	5.12 ± 6.14		25.21 ± 17.52		1.61 ± 0.73	
Divorced	16 (2.9)	4.75 ± 7.71		23.13 ± 13.52		1.81 ± 1.17	
Widowed	21 (3.8)	5.19 ± 5.09	2.884	22.38 ± 12.21	0.219	1.86 ± 0.96	1.361
**Monthly Salary (Yuan)**							
Less than 3000	262 (47.4)	6.15 ± 7.24		26.22 ± 18.44		1.68 ± 0.81	
3000-5000	147 (26.6)	4.89 ± 5.15		27.21 ± 18.09		1.65 ± 0.67	
5000-10000	93 (16.8)	4.10 ± 4.19		22.58 ± 14.06		1.67 ± 0.80	
More than 10000	51 (9.2)	2.69 ± 3.17	11.280^∗^	18.63 ± 12.81	11.405^∗∗^	1.29 ± 0.50	3.835^∗^
**Clinical/Treatment**							
**Cancer Site**							
Breast	468 (84.6)	5.17 ± 5.96		25.34 ± 17.43		1.65 ± 0.76	
Nasopharynx	30 (5.4)	4.97 ± 5.51		24.00 ± 18.31		1.67 ± 0.80	
Colorectal	36 (6.5)	4.39 ± 6.48		23.89 ± 18.09		1.47 ± 0.65	
Leukemia	19 (3.4)	6.32 ± 8.78	2.985	25.26 ± 13.89	1.191	1.53 ± 0.70	0.729
**Stage**							
Stage 1	92 (16.6)	5.57 ± 5.99		29.24 ± 20.23		1.71 ± 0.66	
Stage 2	219 (39.6)	4.37 ± 5.39		24.25 ± 15.64		1.61 ± 0.76	
Stage 3	215 (38.9)	5.80 ± 6.90		24.74 ± 17.95		1.61 ± 0.79	
Stage 4	27 (4.9)	4.85 ± 3.72	3.913	22.22 ± 13.96	4.281	1.67 ± 0.78	0.409
**Daily Exercise Time**							
None	69 (12.5)	6.54 ± 7.15		25.94 ± 16.74		1.74 ± 0.93	
Less than 60 min	427 (77.2)	5.12 ± 6.01		25.20 ± 17.01		1.63 ± 0.73	
60 min and above	57 (10.3)	3.68 ± 4.70	5.514	24.04 ± 20.76	3.135	1.53 ± 0.71	1.261
**Current smoking**							
Yes	21 (3.8)	3.38 ± 4.73		25.24 ± 17.50		1.52 ± 0.60	
No	532 (96.2)	5.22 ± 6.11	–1.602	25.17 ± 17.38	–0.090	1.64 ± 0.76	0.664
**Chemotherapy^a^**							
Yes	501 (90.6)	5.29 ± 6.10		25.49 ± 17.52		1.65 ± 0.76	
No	48 (8.7)	3.96 ± 5.86	–1.703	23.13 ± 16.00	–0.701	1.48 ± 0.65	1.485
**Radiotherapy^a^**							
Yes	479 (86.6)	4.99 ± 5.90		25.28 ± 17.64		1.64 ± 0.76	
No	70 (12.7)	6.43 ± 7.19	–1.521	25.30 ± 15.67	–0.482	1.60 ± 0.75	0.401
**Physical Comorbidity^a^**							
Yes	364 (65.8)	4.70 ± 5.34		24.23 ± 16.54		1.62 ± 0.76	
No	185 (33.5)	6.11 ± 7.27	–1.673	27.35 ± 18.82	–1.755	1.67 ± 0.74	0.804
**BMI**							
Less than 18.5	69 (12.5)	6.13 ± 7.85		22.75 ± 16.53		1.72 ± 0.80	
18.5-25	385 (69.6)	5.05 ± 5.86		25.58 ± 17.82		1.59 ± 0.73	
More than 25	99 (17.9)	4.87 ± 5.44	0.337	25.25 ± 16.18	1.876	1.71 ± 0.82	1.478
**AIS (Insomnia)**							
Yes	225 (40.7)	7.33 ± 6.89		30.00 ± 19.30		1.85 ± 0.86	
No	328 (59.3)	3.65 ± 4.92	–7.682^∗∗∗^	21.86 ± 15.08	–5.219^∗∗∗^	1.47 ± 0.63	–5.905^∗∗∗^
**Psychological**							
**Personality^a^**							
Positive	420 (75.9)	4.77 ± 5.70		25.10 ± 16.76		1.59 ± 0.68	
Negative	129 (23.3)	6.50 ± 7.08	−2.472^∗^	25.89 ± 19.35	–0.20	1.78 ± 0.95	−2.162^∗^
**Number of stressful life event(s)**							
None	136 (24.6)	3.45 ± 4.77		22.87 ± 16.10		1.45 ± 0.61	
Less than 3	212 (38.3)	4.59 ± 5.00		24.58 ± 16.21		1.59 ± 0.70	
3–5	132 (23.9)	6.36 ± 6.27		26.89 ± 18.91		1.80 ± 0.83	
More than 5	73 (13.2)	7.77 ± 8.91	23.397^∗∗∗^	28.08 ± 19.55	4.024	1.77 ± 0.92	6.050^∗∗∗^
**Fear of Progression**							
Yes	165 (29.8)	8.20 ± 7.58		29.94 ± 20.17		1.87 ± 0.88	
No	388 (70.2)	3.85 ± 4.76	–7.331^∗∗∗^	23.14 ± 15.63	–3.527^∗∗∗^	1.53 ± 0.67	–5.013^∗∗∗^
**Depressive symptom**							
Yes	232 (42.0)	7.56 ± 7.27		28.36 ± 18.44		1.84 ± 0.88	
No	321 (58.0)	3.41 ± 4.28	–7.706^∗∗∗^	22.87 ± 16.20	–3.832^∗∗∗^	1.48 ± 0.61	–5.275^∗∗∗^
**Anxiety symptom**							
Yes	170 (30.7)	7.73 ± 7.47		29.41 ± 19.05		1.84 ± 0.85	
No	383 (69.3)	4.01 ± 4.94	–6.150^∗∗∗^	23.29 ± 16.25	–3.748^∗∗∗^	1.54 ± 0.69	–4.303^∗∗∗^

*^*a*^Four participants did not provide relevant data (*n* = 549); ^*b*^Mann-Whitney U test; ^*c*^Kruskal-Wallis test. PRI, pain rating index; VAS, visual analog scale; PPI, present pain intensity; ^∗^*P* < 0.05; ^∗∗^*P* < 0.01; ^∗∗∗^*P* < 0.001.*

### Univariate Analysis

On univariate analysis, low monthly income, insomnia, FoP, depressive, and anxiety symptoms were significantly associated with greater cancer pain (PRI-total, VAS, and PPI subscales). Patients who were older were more likely to report higher scores on PRI-total and VAS. Compared to pessimists, optimists reported lower ratings on PRI-total and PPI. Additionally, we found patients who were experiencing more stressful life events were more likely to report higher levels of pain on PRI-total and PPI (see Table 1).

### Correlations Between Continuous Variables

Table 2 presents the bivariate correlation matrix of continuous study variables. Score of FoP, anxiety, insomnia, and depressive symptoms were significantly associated with different subscales of pain score. The only non-significant correlation in the matrix was between insomnia symptoms and VAS (*r* = 0.052).

**TABLE 2:** Bivariate correlation matrix of continuous study variables.

	**1**	**2**	**3**	**4**	**5**	**6**	**7**	**8**	**9**
1. Fear of Progression	–								
2. Depressive symptoms	0.508^∗∗^	–							
3. Anxiety symptoms	0.480^∗∗^	0.838^∗∗^	–						
4. Insomnia symptoms	0.101^∗^	0.161^∗∗^	0.143^∗∗^	–					
5. PRI-Sensory	0.289^∗∗^	0.338^∗∗^	0.252^∗∗^	0.123^∗∗^	–				
6. PRI-Affective	0.375^∗∗^	0.485^∗∗^	0.423^∗∗^	0.207^∗∗^	0.655^∗∗^	–			
7. PRI-Total	0.343^∗∗^	0.417^∗∗^	0.333^∗∗^	0.162^∗∗^	0.965^∗∗^	0.829^∗∗^	–		
8. VAS	0.218^∗∗^	0.229^∗∗^	0.191^∗∗^	0.052	0.320^∗∗^	0.335^∗∗^	0.352^∗∗^	–	
9. PPI	0.232^∗∗^	0.319^∗∗^	0.250^∗∗^	0.131^∗∗^	0.463^∗∗^	0.450^∗∗^	0.497^∗∗^	0.375^∗∗^	–

*^∗^*P* < 0.05; ^∗∗^*P* < 0.01; PRI, pain rating index; VAS, visual analog scale; PPI, present pain intensity.*

### Hierarchical Regression Model

The corresponding factors that shown significant correlation with cancer pain in previous univariate analyses were then introduced in each regression model (PRI-total, VAS, and PPI model) accordingly as independent variables. Demographic, clinical, and psychological factors were orderly entered in blocks as model 1, 2, and 3. The Durbin-Watson (DW) value of each model, which was a number that tested for autocorrelation in the residuals, was all satisfactory (ranged from 1.820 to 2.184). The variance inflation factor (VIF) (Vatcheva et al., 2016), which quantified the severity of multicolinearity, was all less than 10 (ranged from 1.00 to 3.86).

Tables 3a–c present the results of hierarchical multiple regression of the variables associated with PRI-Total, VAS, and PPI. Among all the tested variables, depressive symptom appeared to have the strongest association with PRI-Total (β = 0.333, *P* < 0.001), VAS (β = 0.159, *P* = 0.043) and PPI (β = 0.301, *P* < 0.001), which demonstrated that those who were depressed were at higher risk of experiencing greater pain. FoP was the second strongest factor associated with PRI-Total (β = 0.231, *P* < 0.001) and VAS (β = 0.142, *P* = 0.004), however, it was not significantly associated with PPI. Insomnia symptom was positively associated with PRI-Total (β = 0.096, *P* = 0.012) and PPI (β = 0.080, *P* = 0.0497), however, it did not significantly correlate with VAS. Age, and anxiety symptom, were not statistically significant in all regression analyses. In all, for each regression model, psychological variables showed stronger association with patient’s pain experience than demographic as well as clinical factors.

**TABLE 3a:** Hierarchical multiple regression of the factors associated with PRI-Total (*n* = 553).

**Factors**	**Model 1**					**Model 2**					**Model 3**				
	**B**	**S.E.**	**Beta**	***t***	***p***	**B**	**S.E.**	**Beta**	***t***	***p***	**B**	**S.E.**	**Beta**	***t***	***p***
Age	0.018	0.023	0.034	0.808	0.419	0.023	0.023	0.042	1.002	0.317	0.042	0.021	0.077	1.966	0.050
Monthly salary	–1.083	0.257	–0.178	–4.217	<0.001	–1.005	0.255	–0.165	–3.942	<0.001	–0.399	0.253	–0.066	–1.576	0.116
Insomnia symptoms						0.074	0.020	0.151	3.633	<0.001	0.047	0.019	0.096	2.521	0.012
Fear of Progression											0.144	0.033	0.231	4.420	<0.001
Anxiety symptoms											–0.110	0.105	–0.073	–1.049	0.295
Depressive symptoms											0.432	0.094	0.333	4.595	<0.001
Personality											–1.032	0.504	–0.094	–2.050	0.041
Number of Stressful events											0.456	0.275	0.073	1.660	0.097
R^2^					0.034					0.057					0.232
Adjusted R ^2^					0.031					0.052					0.220
R^2^ Change					0.034					0.023					0.175
F Change					9.800					13.200					24.731
*P*					<0.001					<0.001					<0.001

*PRI, pain rating index; the Durbin-Watson value of the model was 2.184. The variance inflation factor (VIF) ranged from 1.01 to 3.72.*

**TABLE 3b:** Hierarchical multiple regression of the factors associated with VAS (*n* = 553).

**Factors**	**Model 1**					**Model 2**					**Model 3**				
	**B**	**S.E.**	**Beta**	***t***	***p***	**B**	**S.E.**	**Beta**	**t**	***p***	**B**	**S.E.**	**Beta**	***t***	***p***
Age	0.054	0.066	0.035	0.814	0.416	0.057	0.066	0.037	0.868	0.386	0.078	0.065	0.050	1.197	0.232
Monthly salary	–2.072	0.741	–0.119	–2.795	0.005	–2.006	0.744	0.115	–2.697	0.007	–1.249	0.736	–0.072	–1.697	0.090
Insomnia symptoms						0.063	0.060	0.045	1.059	0.290	0.017	0.059	0.012	0.289	0.773
Fear of Progression											0.253	0.087	0.142	2.916	0.004
Anxiety symptoms											–0.093	0.329	–0.022	–0.283	0.777
Depressive symptoms											0.589	0.291	0.159	2.026	0.043
R^2^					0.016					0.018					0.075
Adjusted R^2^					0.013					0.013					0.065
R^2^ Change					0.016					0.002					0.057
F Change					4.598					1.121					11.162
*P*					0.010					0.290					<0.001

*VAS, visual analog scale; the Durbin-Watson value of the model was 1.820. The variance inflation factor (VIF) ranged from 1.02 to 3.62.*

**TABLE 3c:** Hierarchical multiple regression of the factors associated with PPI (*n* = 553).

**Factors**	**Model 1**					**Model 2**					**Model 3**				
	**B**	**S.E.**	**Beta**	***t***	***p***	**B**	**S.E.**	**Beta**	***t***	***p***	**B**	**S.E.**	**Beta**	***t***	***p***
Monthly salary	–0.078	0.032	–0.103	–2.421	0.016	–0.070	0.032	–0.093	–2.201	0.028	–0.017	0.033	–0.022	–0.503	0.615
Insomnia symptoms						0.008	0.003	0.124	2.926	0.004	0.005	0.002	0.080	1.967	0.0497
Fear of Progression											0.008	0.004	0.097	1.760	0.079
Anxiety symptoms											–0.014	0.014	–0.073	–0.975	0.330
Depressive symptoms											0.049	0.012	0.301	3.903	<0.001
Personality											–0.019	0.067	–0.014	–0.289	0.773
Number of Stressful events											0.039	0.036	0.050	1.085	0.279
R^2^					0.011					0.026					0.120
Adjusted R^2^					0.009					0.022					0.109
R^2^ Change					0.011					0.015					0.094
F Change					5.860					8.559					11.658
*P*					0.016					0.004					<0.001

*PPI, present pain intensity; the Durbin-Watson value of the model was 1.958. The variance inflation factor (VIF) ranged from 1.00 to 3.67.*

## Discussion

The current study is one of the few epidemiological studies that examined the factors associated with cancer patient’s pain experience in mainland China, and it is the first study to investigate the association of FoP and self-perceived pain. In the past, pain was seen only as a biomedical issue. However, nowadays, people have widely recognized the fact that psychological, social as well as environmental factors significantly influence a patient’s pain experience (Gatchel, 2004). Studies revealed that pain experienced over the disease course or during the final cancer stages is not linear or predictable, and an individual’s tolerance levels of pain could be impeded by emotional distress (Elliott, 2014). This highlighted the importance of psychological factors in coping with cancer pain.

In present, pain is considered as the fifth vital sign of a cancer patient’s well-being along with signs of respiration, blood pressure, temperature, and heart rate (Bultz and Carlson, 2006). Our study found that 74.32% of cancer patients in Southern China experience some degree of pain. This figure seemed higher than the rates reported by western researchers which indicated that pain prevalence averaged 50–55% across the continuum from diagnosis through survivorship/end of life (van den Beuken-van Everdingen, 2012; van den Beuken-van Everdingen et al., 2016). One possible reason is that various pain measures were utilized in different study samples, which impedes the direct comparison of results.

Evidence is irrefutable that psychosocial factors play a great role in the relationship between objective pathophysiology and patient’s subjective experience of pain (Vranceanu et al., 2009). As hypothesized, and consistent with existing studies, our study found that anxiety (Novy and Aigner, 2014), and depressive symptoms negatively impact a patient’s self-assessed pain (Cho et al., 2013). Previous studies have reported a high prevalence of depression in patients with chronic pain, and the figure is significantly higher than in the general population and in those without chronic pain (Fishbain et al., 1997; Davison and Jhangri, 2005). Additionally, strong evidence has been provided that depression, anxiety, and FoP were associated with the severity of physical symptoms (Pallant and Bailey, 2005; Chen, 2007, 2018; Koch et al., 2013). The related symptoms of loss of motivation, hopelessness, pessimism, fatigue, and disturbed sleep might be potential reasons to explain patient’s increased pain. Additionally, like kinesiophobia, a significant factor which hinders patient’s rehabilitation and prolongs disability as well as pain (Flanigan et al., 2015), FoP plays a similar role in cancer patient’s recovery and might contribute to the increase and/or maintenance of pain perception.

When coping cancer or other life-threatening diseases, personality is one of the most clearly documented variables (Allison et al., 2000) and has been widely considered as an significant independent variable (Llewellyn et al., 2007). However, in our study, personality was only significantly associated with PRI-total score. One potential reason is that in the current study, personality characteristics was assessed using single-item question, which was insufficient to examine the association in detail. In univariate analysis, patients with more stressful life events were more likely to experience cancer pain. However, the association was no longer significant in further regression analyses. Previous studies assumed that managing stressful life events could largely waste patient’s mental energy and resources (Mellon et al., 2007), therefore, makes them more vulnerable, both psychologically and physically.

Inconsistent with previous findings (Chen, 2007, 2018; Liang, 2016), this study found no relationship between age, gender, cancer stage and patient’s pain experience. Several studies reported that younger patients were more likely to report greater pain as well as pain-related distress (Avis et al., 2012; Cataldo et al., 2013; Krok et al., 2013), however, we found contrary results in our univariate analysis. The reason why we found contradictory results might due to the difference in study sample and participant’s cultural background. Pain issue is multifaceted and complex. Further prospective longitudinal studies are warranted to examine their effects in detail.

### Limitations

There are a number of limitations of the current study. First of all, this study was based on a cross-sectional cohort, the causal relationships between tested variables and cancer pain are still unclear. Longitudinal cohort studies are necessary to identify causal associations. Second, patient’s insomnia, anxiety as well as depressive symptoms were measured only by self-rated questionnaires. No psychiatrist was involved to confirm clinical diagnosis. Also, single item question was used to measure patient’s personality tendency and no psychometric information was provided. Assessment bias may exist and can hardly be eliminated from cross-sectional studies. Further studies using more extensive and objective measurement tools would be beneficial. Third, only hospitals in southern China were included and the majority of the participants were female patients diagnosed with breast cancer. A potential sample bias may exist, and the research findings cannot be generalized to the entire Chinese cancer population. A national study with various cancer populations would be beneficial. Last but not least, more information about the clinical/treatment characteristics of the sample should be included, such as time since diagnosis and surgery type, as those factors may also impact patient’s pain experience.

### Implication

Multidisciplinary groups are necessary in oncology settings to integrate care for pain management. It has been proved by previous meta-analysis and high-quality RCT studies that psychological and behavioral interventions could clinically improve patient’s cancer-related pain (Sheinfeld Gorin et al., 2012; Johannsen et al., 2013). Hypnosis and coping skill training that includes cognitive behavioral therapy components and relaxation with imagery have the strongest evidence in improving pain severity for patients across disease stage (Spiegel and Bloom, 1983; Goodwin et al., 2001). However, these specific pain treatments are inadequate in mainland China. In most of the cases, patients could not be ensured effective pain management (Liang, 2016). Thus, alongside the pharmacological management of pain, psychological pain management should be made more available in health care in China.

## Conclusion

This study demonstrated that insomnia, depressive symptom, and fear of progression in cancer patients generally links to greater self-assessed pain. On the basis of the findings, it is important to evaluate each individual in detail with respect to psychological distress and pain severity when planning treatment and rehabilitation. Multidisciplinary helping groups and relevant coping skill training should be provided to improve patient’s pain management as well as their quality of life.

## Data Availability

The datasets for this manuscript are not publicly available because further articles will be published based on the database. Requests to access the datasets should be directed to 306850475@qq.com.

## Ethics Statement

This study was carried out in accordance with the recommendations of Clinical Research Guideline, Nanfang Hospital Research Ethics Committee with written informed consent from all subjects. All subjects gave written informed consent in accordance with the Declaration of Helsinki. The protocol was approved by the Hospital Research Ethics Committee (Ref No: NFEC-2018-038).

## Author Contributions

YZ and YY: study design. XT, HW, and HS: data collection. WL, TL, and JZ: data analysis and interpretation. YZ, XT, WL, and HW: drafting of the manuscript. BZ and YY: revising of the manuscript. All the co-authors contributed to the approval of the final version for publication.

## Conflict of Interest Statement

The authors declare that the research was conducted in the absence of any commercial or financial relationships that could be construed as a potential conflict of interest.
